# Identification of a rare missense mutation in *GJB1* and prenatal diagnosis in a Chinese family with CMT: A case report

**DOI:** 10.1097/MD.0000000000031733

**Published:** 2022-11-11

**Authors:** Xinyi Huang, Xiaoli Wu, Bei Wu, Jing Mou, Xingwei Ma

**Affiliations:** a School of Medical and Life Sciences, Chengdu University of Traditional Chinese Medicine, Chengdu, China; b Prenatal Diagnosis Center, Guizhou Provincial People’s Hospital, Guiyang, China.

**Keywords:** Charcot-Marie-Tooth disease, *GJB1*, missense mutation, prenatal diagnosis, whole-exome sequencing

## Abstract

Charcot-Marie-Tooth disease (CMT) is a highly heterogeneous genetic disorder. To date, more than 90 genes have been implicated in the pathogenesis of CMT. Here, we report the identification of a rare causative mutation in a Chinese family with CMT and a pregnant patient underwent prenatal diagnosis.

**Patient concerns:**

A 33-year-old woman with 21^ + 6^ weeks of pregnancy presented with progressive weakness of distal extremities after 23 years of age. A total of 8 individuals in 4 generations of her family had similar muscle weakness. On proband whole-exome sequencing (WES), a rare c.121G > A variant in the *GJB1* gene was identified.

**Diagnosis:**

Based on the clinical and genetic findings, this patient was finally diagnosed with CMT.

**Interventions:**

The prenatal diagnosis was performed on the proband fetus.

**Outcomes:**

The fetus did not carry this rare variant, and the pregnancy continued.

**Lessons:**

Our findings provide the first clinical evidence for the causative role of *GJB1* c.121G > A variant in CMT. WES is a valuable method for diagnosing patients with CMT.

## 1. Introduction

Charcot-Marie-Tooth (CMT) is a heterogeneous group of primary genetic neuropathies classically presenting with sensory and motor symptoms. CMT is also known as hereditary sensory and motor neuropathy. Clinically, it is characterized by progressive weakness in the distal muscles, muscle atrophy, pes cavus deformity, sensory loss, diminished tendon reflexes, and reduced nerve conduction velocity.^[1,2]^ Globally, CMT is the most common inherited disorder of peripheral nerves, with a prevalence of 1 in 2500 individuals.^[3]^ Regarding the lack of effective treatment, early genetic assessment and prenatal diagnosis can significantly reduce CMT incidence.^[4]^

CMT has different patterns of inheritance, including the autosomal dominant pattern in CMT1 (demyelinating forms) and CMT2 (axonal forms), the autosomal recessive pattern in CMT4, and the X-linked pattern in CMTX. To date, mutations in more than 90 genes have been implicated in CMT.^[5,6]^ Approximately 90% of CMT cases with a definite genetic diagnosis are caused by only a few of these genes, such as *PMP22*, *GJB1*, *MFN2* and *MPZ*.^[7]^ CMTX alone is the second most common form of hereditary motor and sensory neuropathy, accounting for up to 15% of all CMT cases.^[8]^ CMTX type 1 (CMTX1), constituting 90% of CMTX cases,^[9]^ is caused by mutations in gap junction beta 1 (*GJB1*) gene on chromosome Xq13.1. *GJB1* encodes the connexin 32 (Cx32) protein that forms the gap junction channels in Schwann cells. Loss of Cx32 in the myelinating Schwann cells leads to demyelinating neuropathy.

The definitive genetic diagnosis of suspected CMT cases is based on selective detection of the causative genes. Genes will be selected for detection based on findings from median motor nerve conduction velocity, the pattern of inheritance, and clinical manifestations of the patients. The most common genetic mutation leading to a CMT phenotype is *PMP22* gene duplication/deletion. However, most of CMT cases have mutant variants in other genes, hindering targeted genetic diagnosis.^[10]^ Recently, novel genetic diagnostic methods such as WES facilitated the diagnosis of CMT through the identification of the disease-causing mutation(s).^[11]^ In this study, we identified a rare causative mutation in *GJB1* via WES in a Chinese family with CMT, and conducted the prenatal diagnosis for the proband using Sanger sequencing.

## 2. Case report

**Figure 1.**
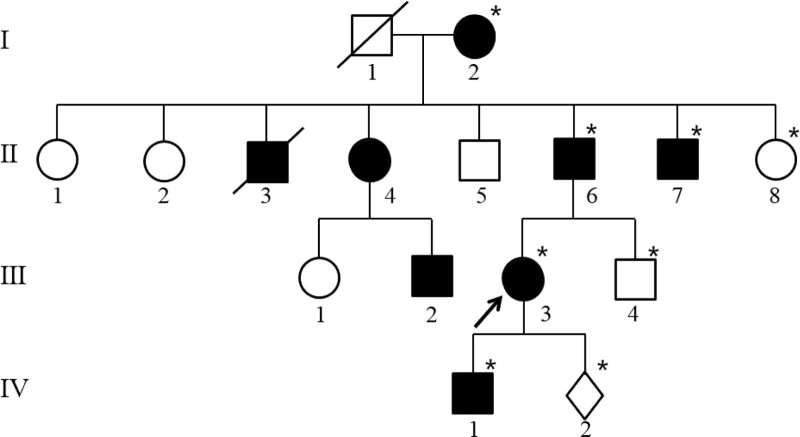
The pedigree of the present family. The proband is indicated by an arrow. Squares indicate men, circles indicate women, diamonds indicate fetus; slashes indicate deceased individuals, shaded (black) symbols indicate individuals with symptoms of CMT, and unshaded symbols indicate individuals without symptoms of CMT. Asterisks indicate members providing DNA samples for genetic analysis. CMT = Charcot-Marie-Tooth disease.

**Figure 2.**
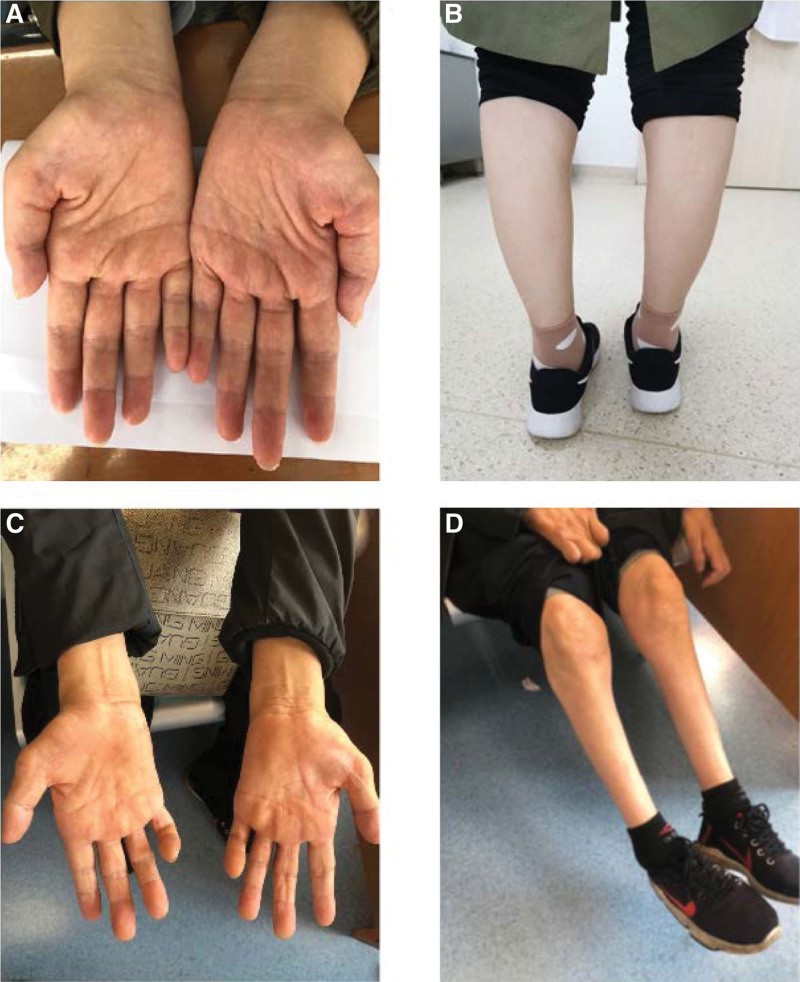
Distal limb muscle atrophy in the proband and her father. The proband presented with atrophy of the thenar and hypothenar muscles, mild atrophy of the gastrocnemius muscle (A and B). The proband’s father had more severe muscle atrophy in his hands and distal to the knees (C and D).

The pedigree is shown in Figure 1. The proband (Ⅲ-3), a 33-year-old Chinese woman, has experienced gradual worsening of muscle weakness in the distal of both upper and lower extremities since 23 years of age. Two years after the onset of symptoms, the proband experienced unsteady gait, easy falls, muscle atrophy in the distal extremities, and foot drop. She did not mention central nervous system symptoms, including cognitive impairment, motor aphasia, dysarthria, dysphagia, and sensory dysfunction. She attended several hospitals and finally received a clinical diagnosis of CMT without a genetic confirmation. At 26, she gave birth to a healthy boy by cesarean section. At 30 years of age, she experienced weakness during walking and was could not run and jump. She visited the Prenatal Diagnosis Center of Guizhou Provincial People’s Hospital for genetic screening and prenatal diagnosis at 21^ + 6^ weeks of gestation of her second pregnancy. She had significant atrophy of the thenar and hypothenar muscles, mild atrophy of the gastrocnemius muscle (Fig. 2A and B). She also had high-arched feet, hallux valgus of both feet, and steppage gait in neurological examinations. The strength of bilateral proximal limb muscles was normal, but distal limb muscles were markedly weak. Her intelligence, orientation, memory, and calculation were normal. Cranial nerve examination showed no pathologic finding. An electrophysiological study revealed the slowing of the motor nerve conduction velocity in the right median (36.88 m/s) and ulnar (37.99 m/s) nerves. Her fetus had no abnormalities on routine prenatal ultrasound screening.

Seven other family members had similar symptoms, most of whom developed gradually increasing distal extremities weakness and muscle atrophy in youth. However, the proband’s paternal grandmother (Ⅰ-2) had a late onset of symptoms and did not experience mild weakness until about 70. In addition, the proband’s paternal grandmother has no significant distal limb muscle atrophy at her 90 years of age. In contrast, the proband’s 56-year-old father (Ⅱ-6) had severe symptoms such as marked atrophy of the thenar and gastrocnemius muscles, claw hand deformity (Fig. 2C and D) and little ability to walk without a walker. The proband’s 7-year-old son (Ⅳ-1) had early-onset symptoms such as unsteady gait and easy falls, but not muscle atrophy. The proband’s mother and 28-year-old younger brother were phenotypically normal. Other members of the pedigree did not visit our department for examination, and their clinical presentation was described by the proband. The electrophysiological study was just performed in the proband.

### 2.1. Genetic study

We collected peripheral blood specimens from 7 members of the family, consisting of 5 symptomatic members, including the proband (Ⅲ-3), her grandmother (Ⅰ-2), father (Ⅱ-6), youngest uncle (Ⅱ-7), son (Ⅳ-1) and 2 asymptomatic members including the proband’s younger brother (Ⅲ-4) and youngest aunt (Ⅱ-8). Three to 5 milliliters of venous blood sample were used for genomic DNA extraction. Under ultrasound guide, an amniotic membrane puncture was performed to obtain fetal exfoliated cells from the proband. A Blood Genomic DNA Kit (Tiangen, Beijing, China) was used to extract DNA from peripheral blood and fetal exfoliated cells according to the manufacturer’s instructions. The DNA concentrations of samples were determined using a NanoDrop 2000 instrument (Thermo, Madison), and then samples were kept at –20°C until WES.

**Figure 3.**
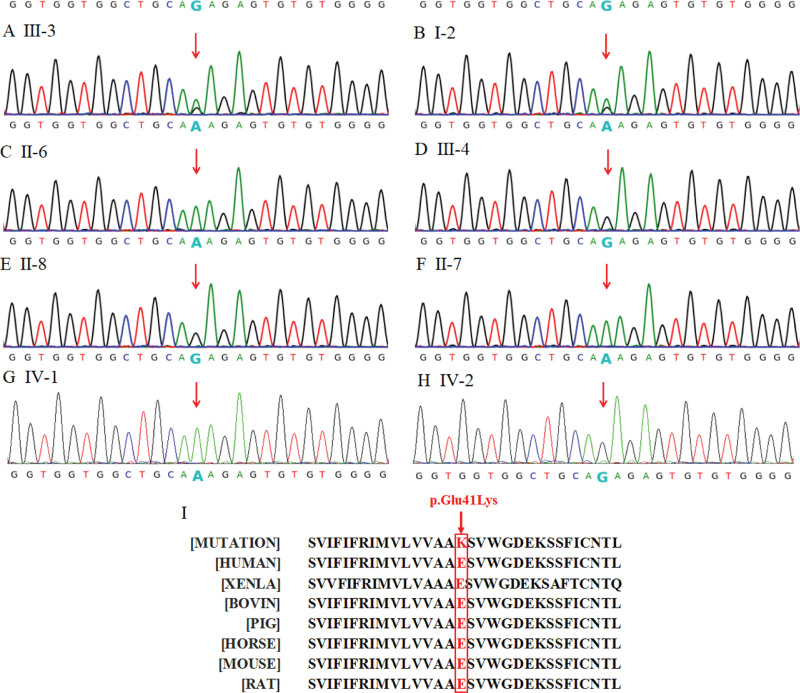
Sanger sequencing and conservation analysis results of the c.121G > A (p.Glu41Lys) variant in the *GJB1* gene. Arrows in the sequencing graphs indicate the position of the c.121G > A variant. The proband (A) and her grandmother (B) are heterozygous for the c.121G > A variant. Proband’s father (C), her son (G), and her youngest uncle (F) were hemizygous for the c.121G > A variant. Proband’s younger brother (D), her youngest aunt (E), and her fetus (H) did not carry the c.121G > A variant. The amino acid affected by c.121G > A (p.Glu41Lys) is highly conserved among 7 species, and the arrow indicates the position of the residues highlighted in red (I).

WES was performed on the sample from the proband. Next-generation sequencing was performed using an Illumina Nextseq 500 sequencer (Illumina, San Diego). The results of WES revealed that the proband carried a heterozygous missense variant in the exon 2 of the *GJB1* gene located on the X chromosome, namely NM_000166.6: c.121G > A (p.Glu41Lys). Sanger sequencing also confirmed the presence of c.121G > A variant in the *GJB1* gene in the proband (Fig. 3A). All female patients examined in the family were heterozygous and the male patients were hemizygous for this variant. Asymptomatic members and the fetus did not carry this variant (Fig. 3B–H). This variant was not found in the East Asian population of the ExAC, 1000 Genomes, and GnomAD database. PolyPhen-2 and SIFT predicted that this variant is probably damaging; MutationTaster predicted that it has disease-causing potential. This variant occurred in the connexion N-terminal functional domain of the Cx32 protein. According to the ACMG/AMP standards and guidelines, the missense variant found in this family was likely pathogenic, and the criteria included PM1, PM2, PP1, and PP3. Although c.121G > A (p.Glu41Lys) was categorized as disease-causing mutation (DM) in the HGMD database, it is an extremely rare mutation, and no cases associated with this variant have been reported so far. Conservation analysis of the mutated amino acid sequence was performed using the Clustal Omega (https://www.ebi.ac.uk/Tools/msa/clustalo/), and revealed that the locus of the missense c.121G > A (p.Glu41Lys) variant was evolutionarily conserved in 7 species (Fig. 3I), suggesting this variant was likely pathological.

This study was approved by the ethics committee of Guizhou Provincial People’s Hospital. Informed written consents were obtained from adult patients and parents in subjects under 18 years of age.

## 3. Discussion

The diagnosis of CMT is mainly based on clinical manifestations, electrophysiological findings, and neuropathological findings. Because of diverse clinical presentations, it is often difficult to make a clinical diagnosis. This diagnostic issue has been recently solved by next-generation sequencing techniques such as WES. Next-generation sequencing techniques are efficient diagnostic methods for detecting known and novel mutations associated with CMT.^[12,13]^ In this study, an extremely rare mutation, c.121G > A (p.Glu41Lys), in the *GJB1* gene was identified by WES in a Chinese family with CMT.

CMTX1, caused by mutations in the *GJB1* gene, is an X-linked dominant CMT. Therefore, there is no father-to-son transmission of CMTX1. Patients with CMTX1 may have typical CMT symptoms such as slowly progressive weakness and atrophy of the distal limb muscles, mostly beginning in childhood or youth. In addition, some patients may present with central nervous system manifestations. The hemizygous male patients carrying the pathogenic mutations are younger and present with more severe clinical manifestations, which will aggravate in older age. About 2-thirds of heterozygous women carrying the pathogenic mutations present with only mild non-progressive symptoms, one-third present with moderate symptoms, and even a small proportion of them do not have any symptoms. The diversity of clinical features may be related to the random inactivation of the X chromosome in the Schwann cells of the peripheral nervous system in women.^[14]^ In this family, the symptoms of the proband and her paternal grandmother were milder than those of the proband’s father. Notably, her grandmother experienced a few mild and late-onset symptoms such as slight muscle weakness. In contrast, the proband’s son experienced severe and early-onset symptoms, such as unsteady gait and easy falls during childhood. The severity of symptoms in different genders in this family was consistent with previous reports in the literature.

The *GJB1* gene encodes the gap junction beta 1 protein connexin 32 (Cx32), expressed by Schwann cells in the peripheral nervous system and by oligodendrocytes and neurons in the central nervous system. Cx32 forms a hexamer in the cell membrane and makes an intact gap junction channel between adjacent cell membranes. Gap junctions provide a direct diffusion pathway for ions and small molecules and conduct rapid communication between the periaxonal and peripheral cytoplasm.^[15]^ Cx32 dysfunction due to pathogenic *GJB1* mutations can be divided into 2 types: absence of Cx32 protein on the cell membrane and presence of Cx32 protein on the cell membrane with an altered gating function. In both cases, the Cx32 protein has impaired or no function, causing damage to peripheral myelin.^[16]^ So far, over 400 mutations have been attributed to *GJB1*. Mutations often result in loss of function rather than gain of function at the protein level.^[17]^

In the family described, we found a rare c.121G > A (p.Glu41Lys) mutation in *GJB1*. Based on a literature review,^[18]^ this variant is classified in the Human Gene Mutation Database (HGMD) as DM. However, this literature review did not provide any detail on clinical manifestations associated with this variant. A different mutation at the same codon (c.123G > C: Glu41Asp) has been reported in a family with CMT. In addition to the common sensorimotor defects, brainstem evoked potential abnormality and white matter lesion on magnetic resonance imaging were reported in this family.^[19]^ However, in this pedigree, we reported that the patients did not have any abnormal manifestations of the central nervous system, and brain magnetic resonance imaging was not performed. Recent studies have denied a direct correlation between the *GJB1* mutations and CMTX disease severity. Even, there was no consistent phenotype-genotype relationship for different mutations at the same codon.^[20,21]^

In addition, c.121G > A (p.Glu41Lys) has been classified as a variant of uncertain significance in the ClinVar database. According to the ACMG/AMP standards and guidelines and findings from this pedigree, we need to reassess the pathogenicity of this variant. This variant is not present in population databases such as ExAC, 1000 Genomes, and GnomAD (PM2). Sanger sequencing showed that the variant co-segregated with CMT within this family (PP1). At codon 41 of *GJB1*, a different variant (c.123G > C) leading to another amino acid replacement (Glu41Asp) has been pathogenic (PM5). Various statistical methods predicted that this variant can harm genes or gene products (PP3). The Cx32 protein has 2 extracellular (EC) loops, which are hot spot mutation domains. Their mutation frequency ranges from 44% in Asian populations to 65% in European populations.^[22]^ This variant occurred in both the connexion N-terminal functional domain, and the first EC loop of the Cx32 protein (PM1). Based on the evidence outlined above, c.121G > A (p.Glu41Lys) variant is classified as a likely pathogenic variant.

There is no specific treatment for CMT, and symptomatic treatment and supportive care, including rehabilitation training, orthopedic surgeries, and symptomatic medication, are the mainstay of treatment. Prenatal diagnosis is currently an effective method to prevent CMT. Therapeutic abortion is recommended when prenatal diagnosis confirms that the fetus is a carrier of a pathogenic mutation in *GJB1*. Amniotic fluid samples analysis by Sanger sequencing showed that the fetus is not a carrier of the c.121G > A (p.Glu41Lys) variant, so the proband safely continued her pregnancy in the aforementioned family.

In the present study, we detected an extremely rare missense mutation, c.121G > A (p.Glu41Lys), in *GJB1* for the first time using WES. In addition, we assessed its pathogenicity for the affected family to be likely pathogenic. Moreover, we performed prenatal diagnosis for the proband’s fetus to help her make the correct decision. Due to clinical and genetic heterogeneity, WES is a helpful tool for diagnosing patients with CMT.

## Acknowledgments

We sincerely thank the patients and their family members for participating in this study.

## Author contributions

**Data curation:** Xinyi Huang.

**Formal analysis:** Xinyi Huang, Xiaoli Wu.

**Investigation:** Xinyi Huang, Bei Wu.

**Methodology:** Xinyi Huang, Jing Mou.

**Project administration:** Xingwei Ma.

**Writing – original draft:** Xinyi Huang, Xiaoli Wu.

**Writing – review & editing:** Xingwei Ma.
